# Comparison of resource use by COPD patients on inhaled therapies with long-acting bronchodilators: a database study

**DOI:** 10.1186/1471-2466-11-61

**Published:** 2011-12-22

**Authors:** Chris M Kozma, Andrew L Paris, Craig A Plauschinat, Terra Slaton, John I Mackowiak

**Affiliations:** 1 CK Consulting, 84 Tomahawk Trail, St. Helena Island, SC, 29920, USA; 2 Vigilytics LLC, 559 Fox Hunt Drive, Victor, NY, 14564, USA; 3 Health Economics & Outcomes Research, Novartis Pharmaceuticals Corporation, 59 Route 10, East Hanover, NJ, 07936, USA; 4 308 Canaberry Drive, West Columbia, SC 29170, USA; 5 Center for Outcomes Research, 3500 Woodmont Blvd, Nashville, TN, 37215, USA

## Abstract

**Background:**

The purpose of this analysis was to compare health care costs and utilization among COPD patients who had long-acting beta-2 agonist (LABA) OR long-acting muscarinic antagonist (LAMA); LABA AND LAMA; or LABA, LAMA, AND inhaled corticosteroid (ICS) prescription claims.

**Methods:**

This was a 12 month pre-post, retrospective analysis using COPD patients in a national administrative insurance database. Propensity score and exact matching were used to match patients 1:1:1 between the LABA or LAMA (formoterol, salmeterol, or tiotropium), LABA and LAMA (tiotropium/formoterol or tiotropium/salmeterol), and LABA, LAMA and ICS (bronchodilators plus steroid) groups. Post-period comparisons were evaluated with analysis of covariance. Costs were evaluated from a commercial payer perspective.

**Results:**

A total of 523 patients were matched using 29 pre-period variables (e.g., demographics, medication exposure). Post-match assessments indicated balance among the cohorts. COPD-related costs differed among groups (LABA or LAMA $2,051 SE = 91; LABA and LAMA $2,823 SE = 62; LABA, LAMA and ICS $3,546 SE = 89; all p < .0001) with the differences driven by study medication costs. However, non-study COPD medication costs were higher for the LABA or LAMA therapy group ($911 SE = 91) compared to the LABA and LAMA therapy group ($668 SE = 58; p = 0.0238) and non-study respiratory medications were approximately $100 greater for the LABA or LAMA therapy group relative to both LABA and LAMA (p = .0018) and LABA, LAMA, and ICS (p = .0071) therapy groups. While there was no observed difference in outpatient costs, there was a slightly higher number of outpatient visits per patient in the LABA and LAMA (25.5 SE = 0.9, p = 0.0070) relative to the LABA or LAMA therapy group (22.3 SE = 0.8) and higher utilization (89.7% of patients) with COPD visits in the LABA and LAMA therapy group relative to both the LABA or LAMA (73.8%; p < .0001) and LABA, LAMA and ICS therapy groups (85.3; p = 0.0305).

**Conclusions:**

Significant cost differences driven mainly by pharmaceuticals were observed among LABA or LAMA, LABA and LAMA and LABA, LAMA and ICS therapies. A COPD-related cost offset was observed from single bronchodilator to two bronchodilators. Addition of an ICS with two bronchodilators resulted in higher treatment costs without reduction in other COPD-related costs compared with two bronchodilators.

## Background

Chronic obstructive pulmonary disease (COPD) is a lung condition characterized by persistent obstruction of bronchial airflow that is not fully reversible. While the disease is progressive, it is possible that treatment may slow the worsening of symptoms.

Treatment includes both pharmacologic and non-pharmacologic alternatives. The Global Initiative for Chronic Obstructive Lung Disease (GOLD) treatment guidelines recommend short acting bronchodilators for mild COPD, adding one or more bronchodilators for moderate to severe COPD, and only adding inhaled steroids for patients with repeated exacerbations [1]. Combining bronchodilators with different mechanisms of action may increase the degree of bronchodilation with equivalent or lesser side effects [2] yet this seems to be a much underutilized form of therapy. The evidence for the clinical efficacy of triple therapy from published randomized controlled clinical trials in patients with moderate-to-severe COPD is limited compared to its application in clinical practice [3]. Regular treatment with inhaled glucocorticoids has been shown to reduce the frequency of exacerbations in patients with severe or very severe COPD [4] with repeated exacerbation [5] however the likelihood of pneumonia increases and mortality is not decreased [6]. Studies have suggested that patients on LABA and LAMA bronchodilator therapy (once daily LAMA plus twice daily LABA) have poor persistence rates, yet instead of dealing with the adherence issues they are often switched to a combination product combining a single bronchodilator and inhaled steroid [7].

COPD treatment goals are to relieve symptoms, prevent disease progression, improve exercise tolerance, improve health status, prevent and treat complications and exacerbations and reduce mortality [1]. There are multiple clinical, humanistic and economic outcomes that are important for the comprehensive evaluation of COPD [8]. These outcomes include variables such as activity level, symptoms, pulmonary function, exercise tolerance, use of rescue medications, satisfaction, quality of life, mortality, and resource use. This study focused on the effect of GOLD guideline recommended pharmacologic treatment on resource utilization and costs, which are outcomes that have a direct impact from a payer perspective.

## Methods

### Study Design

This study was a retrospective, propensity matched-cohort treatment group comparison. An index date was assigned as the date of the first claim for tiotropium, formoterol or salmeterol between January 1, 2006 and June 30, 2007. 12 month pre-index date and 12 month post-index date observation period was used for the evaluation. Randomized trials assume comparison groups are balanced based on random assignment and large sample sizes, whereas retrospective studies frequently use statistical or logical controls to create balanced comparison groups. In this study, a multinomial propensity score matched group approach was employed using patient demographics and other variables from the 12-month pre-index date period. Patients were only included in the analysis if suitable matches were found across the three treatment groups.

### Treatment Group Assignment

The project focused on the long acting inhaled medications listed in Table 1. These products were of interest because they have been shown to relieve symptoms, increase exercise capacity, improve quality of life and reduce exacerbations to a greater extent than short-acting bronchodilators, and today, they are the foundation treatment for this disease [9]. Other therapy combinations with methylxanthines, short acting beta_2_-agonists, short acting anticholinergics, oral or other inhaled glucocorticoids could have been studied, but they were not included in this analysis to minimize the duration of action and delivery route differences in the treatment alternatives.

**Table 1:** Generic names of the long-acting inhaled drug products that were used to assign patients to groups.

Treatment Group	Generic name
LABA OR LAMA*	Formoterol Fumarate
	
	Salmeterol Xinafoate
	
	Tiotropium Bromide

LABA AND LAMA*	Tiotropium Bromide/Formoterol Fumarate
	
	Tiotropium Bromide/Salmeterol Xinafoate

LABA, LAMA AND ICS	Tiotropium Bromide/Fluticasone Propionate/Salmeterol Xinafoate
	
	Tiotropium Bromide/Budesonide/Formoterol Fumarate

* Consistent with GOLD guidelines, refers to bronchodilator therapy.

The following rules were applied to assign patients to treatment groups:

Group 1: LABA or LAMA

a) Patients must have had only claims for one of the study drugs (tiotropium, formoterol or salmeterol) during the 12 month post index period.

b) Patients must have had at least 30 days of medication available in each quarter of the post index year following index to be considered "on therapy".

c) Could not have 30 days or more overlap with any study drugs.

d) Patients using inhaled steroids (budesonide, fluticasone) were excluded

Group 2: LABA and LAMA

a) To qualify for the LABA and LAMA study group, patients must have had prescriptions for either tiotropium in combination with formoterol or tiotropium in combination with salmeterol during the post-index year.

b) Patients must have had at least 30 days of overlapping therapy on two of the drugs during each quarter of the post index year to be considered "on therapy".

c) Patients must not have had any prescriptions for any drug that might make them eligible for the LABA, LAMA, and ICS group during the post-index year.

d) Patients using inhaled steroids (budesonide, fluticasone) were excluded

Group 3: LABA, LAMA, and ICS

a) To be assigned to the LABA, LAMA, and ICS group patients must have had prescriptions for either tiotropium in combination with fluticasone/salmeterol or tiotropium in combination with budesonide/formoterol during the post index year.

b) Patients must have had at least 30 days of overlapping therapy on three of the drugs during each quarter of the post index year to be considered "on therapy".

Once a treatment group was assigned, a patient's first prescription for tiotropium, formoterol or salmeterol was used to establish their index date.

### Study Periods

**Figure 1:**
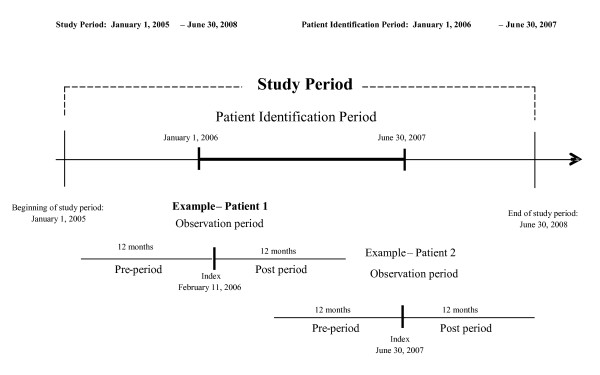
**Graphic representation of the study period and the earliest and latest possible patient observation periods**.

Figure 1 presents a diagram of the study period, illustrating the latest possible index date of June 30^th^, and an earlier potential date of February 11 as examples, showing the requirement for 12 months of data from the pre- and post-index date periods. The utilization data covers the time period from January 1, 2005 through June 30, 2008. A period of one year prior to the index prescription date ("pre-period") was used to apply the inclusion and exclusion criteria and to match patients to form three matched cohorts. Since the available data began on January 1, 2005, the earliest possible date for patient identification was January 1, 2006. This identification period continued until June 30, 2007, which allowed one full year of follow up observation ("post-period").

### Inclusion/Exclusion Criteria

The following inclusion and exclusion criteria were employed to ensure that study patients were accurately identified as COPD patients, were not compromised by other respiratory conditions, and had complete claims information during the study period.

#### Inclusion Criteria

The following criteria, all of which must have been satisfied, were used to identify patients for this study:

• Have at least one diagnosis for COPD (490.xx-492.xx,494.xx,496.xx) in any diagnosis field anytime in the available data.

• Have a prescription claim for one of the study medications between Jan 1, 2006 - June 30, 2007. The date of the first study medication is the index date.

• Have at least 30 days of medication per quarter in the 12 months post-index meeting the criteria described under study group assignment.

• Have continuous enrollment for 12 months pre and 12 months post index date. Be at least 40 years of age on index date.

#### Exclusion Criteria

If any one of the following criteria was met, the patient was excluded from the study.

• Incomplete or invalid data on variables that were used in the either the propensity-match analysis (e.g. age, gender) or post-index analysis (e.g. costs).

• Have a diagnosis of asthma (ICD-9-CM 493.xx), cystic fibrosis (277.0x), or respiratory tract cancer (160.x-164.x) at any time in the available data.

• Missing or zero quantity or days supply for any study drug claims in the Study Period.

### Propensity Match Procedure

After patients were assigned to the three treatment groups, and after the application of the inclusion and exclusion criteria, a 1:1:1 propensity match was conducted to provide evidence of equivalence between the comparison groups. A multinomial logit propensity model was employed with a methodology published by Rosenbaum and Rubin [10] and extended to more than two groups by Imbens [11]. Our approach used exact matching and nearest available Mahalanobis distance matching within calipers defined by the propensity scores. The process created three treatment groups that were closely matched on the pre-index date characteristics (see results tables for a listing of the variables included in the analysis). Exact matching was performed on 4 of these variables (gender, south region, pneumonia and ischemic heart disease). Mahalanobis distance matching within calipers for the propensity score was used to select the best matching patients using the remaining variables. To account for correlation that may have been introduced by matching patients, paired tests were used to test for any differences remaining within the matched variables. Independent tests were also evaluated since they provide additional information on the mean effects. Since the results were similar and the paired tests are more conservative, only the results of the paired tests are presented.

Dichotomous indicators were included in the propensity model for most frequently reported physician types (e.g., cardiovascular specialist and internists) and for specific comorbidities based on presence of diagnosis codes (e.g., neoplasms, pneumonia, hypertension, heart failure, respiratory illness, diabetes, ischemic heart disease, pulmonary vascular disease and stroke). Use of the Charlson comorbidity index was evaluated, however, better model fit was observed with the specific comorbidity indicators and the prescription measure (i.e., the Rx comorbidity score). The prescription measure is a continuous variable that is the count of number of different medication classes (i.e., treated comorbidities) from which patients had prescriptions dispensed. The propensity model also includes the percentage of days in the pre-period for which patient had COPD medications available. This was assumed to be a measure of pre-period medication adherence with COPD medications. Utilization and cost variables were included in the propensity model (e.g., presence of a hospitalization or ER visit in the pre-period, COPD-related total cost in the pre-period, number of hospital visits, number of hospitalized days, number of ER visits, and number of outpatient visits). Finally, an indicator for payer status was included in the model (i.e., Medicare and other plan type). See results tables for a listing of the variables included in the analysis

### Outcome Comparisons

A generalized estimating equations (GEE) model with repeated measures was used to conduct analyses of covariance tests for differences in primary outcomes among the three propensity matched groups. This method was selected to enable adjustment of potentially higher correlation between matched patients compared to non-matched patients. The model utilized the normal distribution and an exchangeable covariance matrix. Use of the gamma distribution was evaluated; however, the normal distribution provided better model fit. A variable for days of study medication exposure during the year prior to Index date was included as a covariate in all outcome models. Differences in least-square mean costs and utilization among treatment groups were then tested.

The primary study outcomes, health care costs and utilization rates, were compared for the three propensity matched cohorts during the 12 month post-index date period. Total costs were evaluated as well as costs by type of service and COPD relatedness (i.e., COPD-related or non-COPD related). Cost was evaluated from a commercial insurer perspective and included amounts that would typically be reimbursed by a commercial payer. Types of service included inpatient services, emergency room services, outpatient services, and pharmacy. Utilization rates (e.g. visits/year) were also summarized overall and by service category.

Post-index date comparisons among the matched groups were conducted using a) GEE models adjusting for repeated measures with follow-up pairwise tests for each continuous variable or b) Cochran's Q tests with follow-up pairwise McNemar's tests for categorical variables. "Days of study medication exposure" during the pre-index date year was used as a covariate in the post-period comparisons. To make the costs incurred across the study period comparable, the consumer price index (CPI) was used to bring all costs to June, 2008 using the US city average medical care services CPI (not seasonally adjusted) to escalate earlier costs to June, 2008 [12]. The data source for this study was the MarketScan database, a national managed care claims dataset. The data was selected from the MarketScan database from January 1, 2005 through June 30, 2008. SAS Version 9.1 was used for all analyses. No adjustments were made for multiple comparisons as part of the propensity analyses. The Marketscan data are irreversibly de-identified and according to the Health Insurance Portability and Accountability Act of 1996, no institutional review board approval or waiver of authorization was required.

## Results

Of the 49 million unique patients in the database during the study period, 163,174 used at least 1 of the COPD study drugs, and 15,857 met the remaining inclusion and exclusion criteria which qualified them to be assigned to one of the three treatment groups as shown in Table 2. The majority of patients were excluded based on diagnoses, eligibility and minimum drug exposure requirements. The starting size of the sample that had diagnosed COPD without asthma, which were continuously eligible for services over the study period and had minimum exposure to study medications, was 26,245. Of these, 16,556 had one of the drug patterns of interest and after exclusions for missing enrollment information and negative dollar amounts the final sample size was 15,857. A total of 592 were assigned to the LABA and LAMA group providing evidence that this therapy is underutilized based on the recommendations in the GOLD guidelines.

**Table 2:** Attrition of patients using the inclusion/exclusion criteria and assignment to three treatment groups before 1:1:1 matching process.

Criteria	Removed	Remaining
Number of patients MarketScan database (1/2005-6/2008)	NA	49,042,666

Have at least one COPD Study Drug between Jan 1, 2006 - Jun 30, 2007)	48,879,492	163,174

Omit patients with missing or zero quantity or days supply values on any COPD study prescription claim	2,785	160,389

Require at least one diagnosis for COPD (490.xx-492.xx, 494.xx, 496.xx) in any diagnosis field at any time in the data.	44,560	115,829

Omit patients with RX = 0 on any claims medical during the study period; this is MarketScan's indicator that the pharmacy claims were incomplete or unavailable.	690	115,139

Omit patients with a diagnosis of asthma (ICD-9-CM 493.xx), cystic fibrosis (277.0x), or respiratory tract cancer (160.x-164.x) at any time during the available data.	51,149	63,990

Require 12 months pre/12 months post index continuous eligibility	19,871	44,119

Include only patients aged 40 or older on index date	205	43,914

Have at least 30 days per quarter coverage for index prescription	17,669	26,245

Have at least one prescription for tiotropium, formoterol or salmeterol with treatment patterns that were in keeping with one of the three treatment groups (LABA or LAMA, LABA and LAMA or LABA, LAMA and ICS therapy)	9,689	16,556

Omit patients with missing PLANTYPs in CCAE enrollment data	55	16,501

Omit patients with negative dollar amounts in the NETPAY field.	644	15,857

TOTAL		**15,857**

		

**Treatment group distribution prior to propensity match:**	**n**	**%**

LABA or LAMA group	8,334	52.6

LABA and LAMA group	592	3.7

LABA, LAMA, and ICS group	6,931	43.7

### Propensity Match

Of the 592 patients in the LABA and LAMA group, a 1:1:1 match was made for 523 of the patients. The remaining patients did not have a suitable match in the other groups for inclusion into the 3 matched cohorts. Tables 3 and 4 show the comparability of the three matched cohorts on the 29 pre-index date match variables. In addition, plots of the propensity score logits were visually inspected and showed extensive overlap with similar distributions across cohorts (data not shown).

**Table 3:** Comparison of three matched cohorts on their pre-index date categorical variables using McNemar's test for paired observations.

	LABA or LAMATx (n = 523)		LABA and LAMA Tx (n = 523)		LABA, LAMA, and ICS Tx (n = 523)		Significant Pairwise tests*
	
Categorical Variables	n	(%)	n	(%)	n	(%)	
Male	280	(53.5)	280	(53.5)	280	(53.5)	NONE

North	210	(40.2)	218	(41.7)	222	(42.4)	NONE

Northeast	52	(9.9)	50	(9.6)	49	(9.4)	NONE

South	189	(36.1)	189	(36.1)	189	(36.1)	NONE

West	71	(13.6)	65	(12.4)	61	(11.7)	NONE

Cardiovascular specialist	170	(32.5)	181	(34.6)	176	(33.7)	NONE

Internist	253	(48.4)	260	(49.7)	264	(50.5)	NONE

Neoplasms	128	(24.5)	146	(27.9)	149	(28.5)	NONE

Pneumonia	51	(9.8)	51	(9.8)	51	(9.8)	NONE

Hypertension	187	(35.8)	209	(40.0)	203	(38.8)	NONE

Heart failure	59	(11.3)	53	(10.1)	56	(10.7)	NONE

Diseases of the respiratory system	482	(92.2)	475	(90.8)	477	(91.2)	NONE

Diabetes	67	(12.8)	71	(13.6)	73	(14.0)	NONE

Ischemic heart disease	116	(22.2)	116	(22.2)	116	(22.2)	NONE

Pulmonary vascular disease	16	(3.1)	19	(3.6)	15	(2.9)	NONE

Stroke	40	(7.6)	45	(8.6)	37	(7.1)	NONE

Hospital visit (≥ 1)	89	(17.0)	81	(15.5)	68	(13.0)	NONE

ER visit (≥ 1)	148	(28.3)	142	(27.2)	129	(24.7)	NONE

ER visit leading to hospitalization (≥ 1)	62	(11.9)	53	(10.1)	55	(10.5)	NONE

Non-Medicare payer type**	156	(29.8)	146	(27.9)	161	(30.8)	NONE

*Based on McNemar's tests for paired observations. Pairwise tests were conducted and results are listed using the following notation: M = LABA or LAMA, D = LABA and LAMA, T = LABA, LAMA, ICS, NONE = No sig dif. All variables were derived from the 12 month pre-index period.

**An indicator variable for Medicare versus Non-Medicare plan type was used. The plan type of Non-Medicare included Comprehensive, HMO, POS, EPO, PPO, and CDHP.

**Table 4:** Comparison of three matched cohorts on their pre-index date continuous match variables using paired t-tests.

Pre-Index Continuous Variables	LABA or LAMATx		LABA and LAMA Tx		LABA, LAMA, and ICS Tx		Significant Pairwise Paired t-tests*
	(n = 523)		(n = 523)		(n = 523)		
	
	LS Mean (SE)		LS Mean (SE)		LS Mean (SE)		
Age	70.0	(9.4)	70.7	(9.4)	70.4	(9.3)	NONE

Rx comorbidity score	6.2	(2.6)	6.4	(2.8)	6.2	(2.6)	NONE

% Days COPD medication exposure	64.3	(32.3)	66.3	(33.8)	63.2	(32.8)	DvT

Average copay amount	12.9	(12.4)	13.9	(13.4)	14.0	(12.4)	NONE

Total COPD-related costs	1,895.9	(1914.5)	1,987.7	(2411.5)	1,973.1	(2216.6)	NONE

Number of hospital visits	0.2	(0.5)	0.2	(0.5)	0.2	(0.5)	NONE

Number of days in hospital	0.8	(2.5)	0.8	(2.3)	0.6	(2.3)	NONE

Number of emergency room visits	0.5	(1.1)	0.4	(1.0)	0.4	(0.8)	NONE

Number of outpatient visits	21.7	(16.8)	22.4	(16.7)	20.9	(18.8)	NONE

*Pairwise paired t-tests for dependent measures were conducted and pairs resulting in a significant p-value are listed (M = LABA or LAMA, D = LABA and LAMA, T = LABA, LAMA, ICS, NONE = No sig dif). All variables were derived from the 12 month pre-period.

### Outcome Comparisons

**Figure 2:**
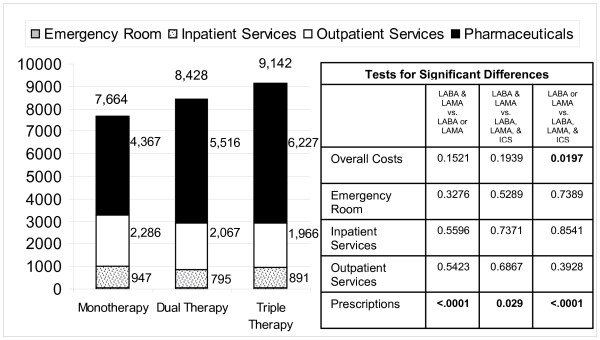
**Average overall health care costs per patient in each matched treatment cohort**. (n = 523 patients per matched cohort) (Emergency Room costs are not visible due to the very small magnitude).

When the three matched cohorts were compared on the outcome of total commercial paid amounts, the LABA, LAMA, and ICS cohort with a cost of $9,142/year on average, had significantly higher costs versus the LABA or LAMA group at $7,664 on average (p = 0.0197). When total prescription costs were compared statistically, all 3 groups differed from one another significantly. The incrementally higher expenditures for each successive level of therapy did not result in an overall health care cost savings or offset when costs were viewed in the aggregate as shown in Figure 2.

The same pattern was observed when COPD-related costs were compared, which also differed among groups (LABA or LAMA $2,051 SE 91; LABA and LAMA $2,823 SE 62; LABA, LAMA and ICS $3,546 SE 89; all p < .0001) with the differences driven primarily by study medication costs.

**Figure 3:**
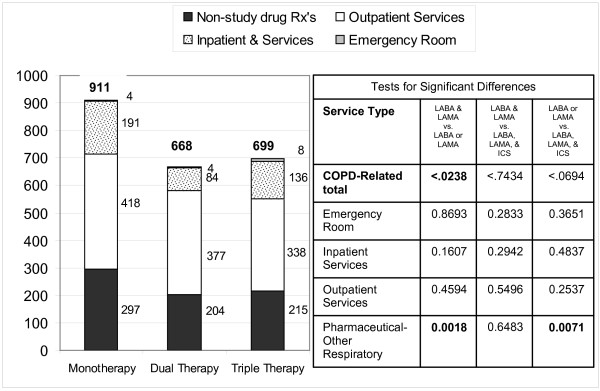
**Average COPD related expenditure excluding the expenditure the study drugs (monotherapy, dual therapy or triple therapy**.) Totals are shown at top of each bar. Service type subtotals shown next to bar. (Emergency Room costs are not visible due to the very small magnitude).

Partial offset of the COPD-related cost was observed when expenditures were classified by service type and their relationship to COPD vs. non-COPD expenditure as shown in Figure 3 with study drug costs not shown. The matched cohort treated with LABA or LAMA had significantly higher COPD-related expenditures (after study drug was excluded) compared to the matched cohort treated with LABA and LAMA therapy. The expenditure for other respiratory prescriptions (albuterol, theophylline, etc.) was also higher in the LABA or LAMA cohort compared to the LABA and LAMA or LABA, LAMA and ICS therapy cohorts (p = 0.0018 and 0.0071 respectively). The LABA or LAMA cohort also trended to have higher expenditures for inpatient and outpatient services, however these differences were not statistically significant when then these service types were analyzed separately.

Paradoxically, while expenditure trended higher in the mono therapy group for outpatient service, the percent of patients using outpatient services (73.8%) was lower than in the LABA and LAMA therapy cohort (89.7%, p < 0.0001) and the LABA, LAMA and ICS therapy cohort (85.3%, p < 0.0001). In addition, the number of outpatient visits per patient per year was also lower in the LABA or LAMA cohort (22.3 visits/year) compared to the LABA and LAMA therapy cohort (25.5 visits/year, p < 0.0001); but not significantly different than the LABA, LAMA and ICS therapy cohort (23.5 visits/year, p = 0.2700). When only COPD-related visits are compared, the LABA and LAMA therapy cohort used 8.5 COPD-related visits/year, compared to the LABA, LAMA and ICS therapy cohort using 7.3 COPD-related visits/year (p = 0.0355).

## Discussion

This study using existing retrospective data employing a statistical method to identify matched cohorts of patients compared the matched cohorts on the pre-specified economic outcomes. Such methods are not likely to replace the randomized clinical trial, yet they have other advantages in that large samples of patients can be studied in real-world settings using existing data.

Even before any comparisons were made between cohorts, an important finding was that only 3.7% of all the COPD patients assigned to a treatment group were using combination long-acting inhaled beta agonist and antimuscarinic bronchodilator therapy, in spite of the fact that this regimen is recommended by GOLD before adding ICS therapy. This low percentage may indicate that COPD patients do not stay on LABA and LAMA therapy for long before they are elevated to LABA, LAMA and ICS therapy, or that patients may progress directly from LABA or LAMA to LABA, LAMA and ICS therapy.

Although the LABA and LAMA therapy group was small compared to the size of the other 2 groups, the matching process successfully resulted in three very similar cohorts using the pre-index date characteristics available in the data. If FEV-1 or other clinical endpoints were available, the match between cohorts might have been further enhanced, however those data elements are not available in the administrative claims dataset used in this study.

As the number of COPD treatments increased from the single to the LABA, LAMA and ICS therapy cohorts, the cost of prescription treatment increased; and there was a partial offset in COPD related costs between the LABA or LAMA group and the LABA and LAMA therapy group. Specifically, the total health care costs were on average $1149 higher in the LABA and LAMA therapy group compared to the single therapy group, and the COPD-related costs were $243 lower, excluding the cost of the study drugs. A similar offset from other COPD-related costs was not seen when the LABA and LAMA therapy group was compared to the LABA, LAMA and ICS therapy group. While other clinical or quality-of life outcomes might have been improved in the LABA, LAMA and ICS therapy cohort, the economic outcomes as measured by the administrative claims data did not improve in the LABA, LAMA and ICS therapy cohort.

The LABA or LAMA cohort had higher COPD related cost, however the number of outpatient visits and the percent of patients with visits were significantly lower. One potential explanation of this observation was that the visits within the LABA or LAMA cohort were more expensive per visit, possibly indicating they were for treatment of exacerbations, and not for routine visits.

Because many patients were excluded in the match selection process, these findings should not be taken to represent what would be seen in all COPD patients. The findings pertain to the matched cohorts which include the LABA and LAMA therapy cohort. This indicates the need for additional research regarding the potential benefits of maximizing LABA and LAMA therapy before moving a COPD patient to LABA, LAMA and ICS therapy with inhaled glucocorticoids. A 1:1:1 propensity match was used in this analysis. It is possible that alternative selection or matching strategies could lead to different results. Further exploration using alternative techniques is warranted.

While the goal of the study was to evaluate patients who are on a stable therapy, assignment of patients to static groups may make detection of differences in utilization less likely. Little is known about the process that physicians use when deciding to make changes in patient medications; however it might be reasonable to expect that changes are more likely to occur when patients are having problems and that stable patients have lower levels of utilization. Follow-up studies may focus on the transitions or switches in therapy among the LABA or LAMA, LABA and LAMA, or LABA, LAMA and ICS therapy options.

The use of claims based data for conducting propensity matching is limited to the variables that are included in an administrative database. No objective clinical measures of COPD severity or functional status were available. While the 29 variables used in the match process indicated the 3 cohorts were very well matched, the potential exists that some other characteristic not captured by the 29 matched variables may explain a difference between the 3 cohorts at baseline.

As the health care system works to achieve meaningful use of electronic medical records, studies such as this may become more common and more advanced as we gain insights to improve health care quality and/or lower cost by analyzing real-world data. Incorporating clinical outcomes and functional status into an analysis such as this could provide additional benefits.

## Conclusions

The findings from this analysis of administrative data are consistent with the randomized trial findings and recommendations within the GOLD COPD treatment guidelines. The use of LABA or LAMA alone may result in higher COPD-related management costs compared to managing stable patients with a LABA and LAMA regimen or a LABA, LAMA and ICS regimen. This study also indicates that a stable COPD population managed with LABA, LAMA and ICS regimen including inhaled corticosteroids may have higher treatment costs without an offset in their other COPD-related costs compared to a matched cohort managed with a LABA and LAMA regimen. Because no information on clinical or quality of life outcomes were assessed, it is not possible to report the effect of additional levels of COPD therapy on these non-economic outcomes.

## Competing interests

CK, AP, TS, and JM are consultants whose services are funded by Novartis. CP is an employee of Novartis.

## Authors' contributions

CK and TS participated in the design of the study and performed the statistical analysis.

AP and CP conceived of the study, and participated in its design and coordination and helped to draft the manuscript.

JM drafted the manuscript and developed the tables and figures

All authors read and approved the final manuscript.

## Pre-publication history

The pre-publication history for this paper can be accessed here:

http://www.biomedcentral.com/1471-2466/11/61/prepub
